# LncRNA PVT1 facilitates the growth and metastasis of colorectal cancer by sponging with miR‐3619‐5p to regulate TRIM29 expression

**DOI:** 10.1002/cnr2.2085

**Published:** 2024-06-04

**Authors:** Zhenni Sun, Xutong Li, Yanyan Shi, Yasai Yao

**Affiliations:** ^1^ Department of Oncology, Qingdao Municipal Hospital Medical College of Qingdao University Qingdao Qingdao Shandong People's Republic of China; ^2^ Department of Oncology Qingdao women and children's Hospital Qingdao Shandong People's Republic of China; ^3^ Department of Medical oncology Qingdao Fuwai Cardiovascular Hospital Qingdao Shandong People's Republic of China

**Keywords:** colorectal cancer, epithelial‐mesenchymal transition (EMT), lncRNA plasmacytoma variant translocation 1 (PVT1), miR‐3619‐5p, tripartite motif containing 29 (TRIM29)

## Abstract

**Background:**

Colorectal cancer (CRC) is the second most common cause of cancer‐related death worldwide. Long noncoding RNA (lncRNA) is involved in many malignant tumors. This study aimed to clarify the role of the lncRNA plasmacytoma variant translocation 1 (PVT1) in CRC growth and metastasis.

**Methods:**

Differentially expressed lncRNAs in CRC were analyzed using the Cancer Genome Atlas. Gene expression profiling interactive analysis and a comprehensive resource for lncRNAs from cancer arrays databases were used to analyze lncRNA PVT1 expression and CRC prognosis, respectively. Cell counting kit‐8, wound healing, colony formation, Transwell, and immunofluorescence assays were used to evaluate CRC cell proliferation, migration, invasion, and epithelial‐mesenchymal transition (EMT), respectively. Tumor growth and metastasis models were used to explore the PVT1 effect on the growth and metastasis of CRC in vivo.

**Results:**

PVT1 was highly expressed in CRC, associated with a poor prognosis of CRC, and showed good diagnostic value. Transfection of sh‐PVT1 or pcDNA3.1‐PVT1 reduced or increased the proliferation, wound healing rate, colony formation, invasion, and EMT of CRC cells. PVT1 and miR‐3619‐5p were co‐expressed in CRC cytoplasm, and PVT1 acted as a competitive endogenous RNA (ceRNA) by sponging miR‐3619‐5p to up‐regulate tripartite motif containing 29 (TRIM29) expression. MiR‐3619‐5p overexpression and TRIM29 knockdown reduced proliferation, wound healing rate, invasion, and EMT of CRC cells. However, simultaneous PVT1 and miR‐3619‐5p overexpression or knockdown of miR‐3619‐5p and TRIM29 knockdown rescued the malignant phenotype of CRC cells.

**Conclusions:**

We first clarified the ceRNA mechanism of PVT1 in CRC, which induced growth and metastasis by sponging with miR‐3619‐5p to regulate TRIM29.

## 1 INTRODUCTION

Colorectal cancer (CRC) is among the most common malignant tumors in humans and is the leading cause of cancer‐related deaths worldwide after lung cancer.^1^, ^2^ Nearly 90% of CRC‐related deaths are caused by distant metastasis of cancer cells, primarily liver metastasis.^3^ Although clinical diagnosis and treatment have improved and prolonged patient survival, the survival prognosis of patients with metastatic CRC remains poor. During metastasis, cancer cells change from an epithelial to a mesenchymal phenotype. This process is called epithelial‐mesenchymal transition (EMT).^4^, ^5^ EMT causes cancer cells to leave the primary tumor site, invade surrounding tissues, and migrate to distant organs. These cells become epithelial again after planting and develop metastases.^4^, ^6^ Consequently, the molecular pathways governing EMT must be investigated to reduce CRC metastasis and develop effective CRC treatments. Increasing evidence shows that long noncoding RNAs (lncRNAs) are involved in malignant tumor development.^7^, ^8^ LncRNAs are transcripts greater than 200 nucleotides in length and have limited protein‐coding potential. Several studies have shown that lncRNAs regulate epigenetic, transcriptional, and posttranscriptional processes to participate in cancer development.^9^, ^10^ Currently, tens of thousands of lncRNAs have been identified, many uniquely expressed in differentiated tissues or specific cancer types.^11^ Different cellular locations of lncRNAs lead to different regulatory processes. Nuclear lncRNAs are involved in chromatin interactions, transcriptional regulation, and RNA processing, whereas cytoplasmic lncRNAs regulate the stability or translation of mRNAs and affect the cell signal transduction cascade.^9^, ^12^ It has been widely proposed that lncRNAs act as micro‐RNA (miRNA) competitive endogenous RNA (ceRNA) through their binding sites to regulate miRNA target gene expression, thereby participating in regulating tumor malignant phenotypes.^13^, ^14^, ^15^ For example, lncRNA‐CDC6 as ceRNA promotes the proliferation and metastasis of breast cancer cells in vivo and in vitro through the microRNA‐215/CDC6 axis.^16^ LINC01133, a ceRNA, regulates adenomatous polyposis coli expression and the Wnt/β‐catenin pathway through sponging miR‐106a‐3p to inhibit gastric cancer development and metastasis.^17^ MiR‐3619‐5p has been reported to be involved in regulating the malignant progression of gastric cancer,^18^ lung cancer,^19^ and gallbladder cancer,^20^ and is associated with cancer cell proliferation, migration, invasion, and apoptosis. However, the molecular mechanism of plasmacytoma variant translocation 1 (PVT1) targeted regulation of miR‐3619‐5p in CRC is not yet clear. The tripartite motif‐containing 29 (TRIM29), an ubiquitin E3 ligase, is linked to cancer development. In papillary thyroid cancer (PTC), it was found that TRIM29 can promote PTC cell proliferation, colony formation, and cell cycle.^21^ CRC also found that TRIM29 can regulate glucose metabolism to promote CRC cell malignancy.^22^ TRIM29 also affects the CRC cell migration, invasion ability^23^ and EMT.^24^ However, the role of lncRNAs as ceRNAs in CRC development and metastasis remains unclear. The mechanism by which the lncRNA PVT1 regulates the miR‐3619‐5p/TRIM29 axis in CRC development and metastasis has not yet been reported.

**FIGURE 1:**
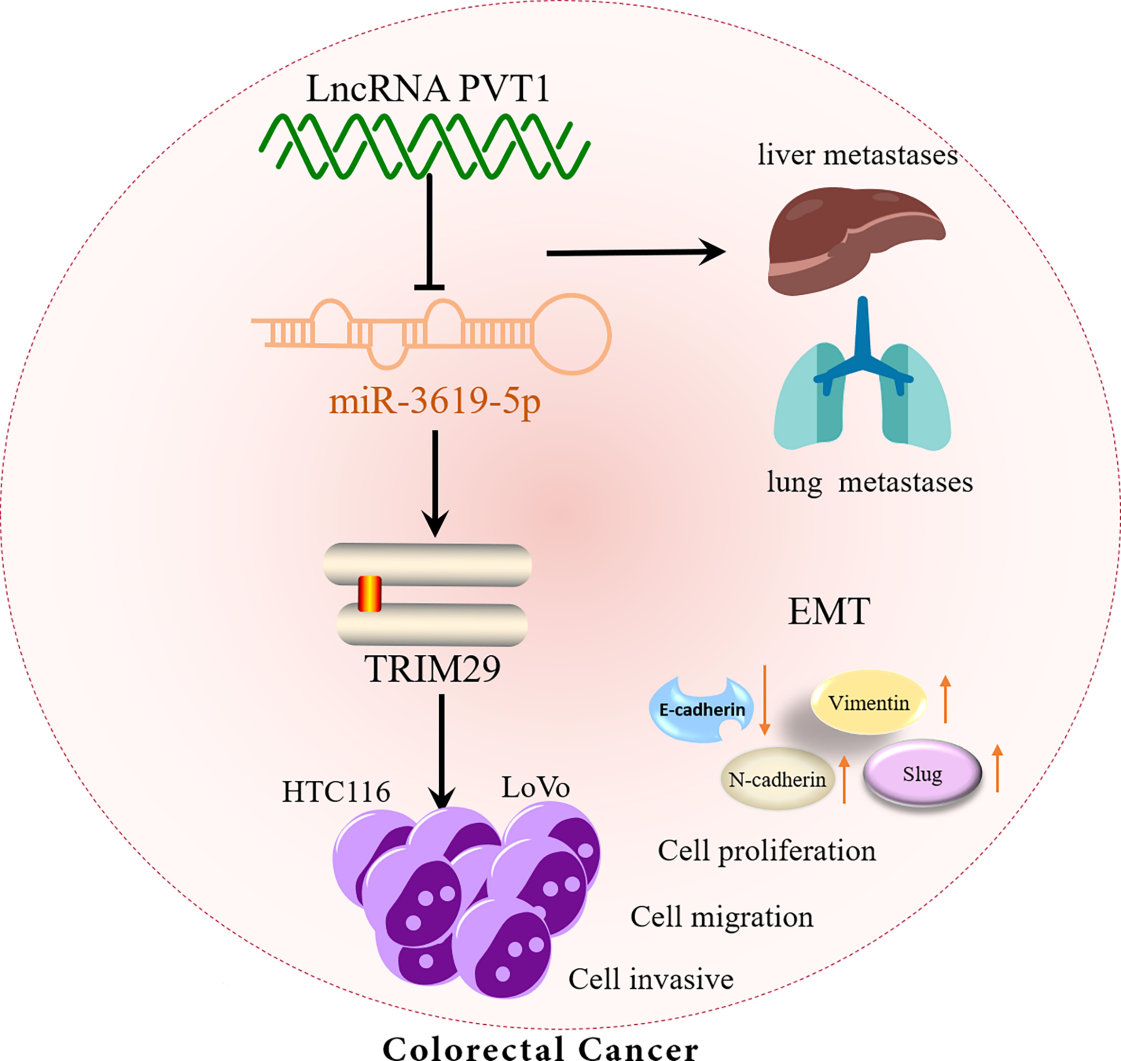
LncRNA PVT1 facilitates colorectal cancer graphical abstract. PVT1, plasmacytoma variant translocation 1.

In this study, we found that PVT1 was highly expressed in CRC based on the analysis of the Cancer Genome Atlas (TCGA) database, which was associated with poor prognosis and had good diagnostic value. Functional assays demonstrated that PVT1 promotes CRC proliferation and metastasis in vivo and in vitro by sponging miR‐3619‐5p to regulate TRIM29. It is suggested that PVT1 has carcinogenic potential and is very likely to be a potential target for CRC diagnosis and treatment Figure 1.

## 2 MATERIALS AND METHODS

### 2.1 Clinical tissues and cells

Between 2018 and 2020, 27 pairs of human CRCs and paired adjacent tissues were collected. Para‐cancerous CRC tissues were obtained at a distance of at least 3 cm. None of the patients had undergone radiotherapy or chemotherapy before surgery. All CRC tissues were subjected to a reverse transcription‐quantitative polymerase chain reaction (RT‐qPCR), and the high and low expression of PVT1 were divided by the median value. Then, correlation analysis was performed. Patient information is presented in Table 3. All patients signed an informed consent form. The research complied with the principles of the “Declaration of Helsinki.”

Human normal colonic epithelial cells (NCM460), human CRC cell lines (HCT116, HT‐29, LoVo, SW480, and LS174T), and mouse colonic cancer cells (CT26.WT) were purchased from the Institute of Biochemistry and Cell Biology, Chinese Academy of Sciences (Shanghai, China). HCT116 and LoVo cells were incubated in McCoy's 5A (16 600 082, Thermo Fisher Scientific, USA) and F12k (21 127 030, Thermo Fisher Scientific, USA) media, respectively, each containing 10% fetal bovine serum (FBS). The remaining cells were cultured in Dulbecco's modified eagle medium (DMEM) (HyClone, South Logan, UT, USA) containing 10% FBS, 100 U/mL penicillin, and 100 mg/mL streptomycin and were cultured in a 5% CO_2_ cell incubator at 37°C.

### 2.2 Plasmid construction and cell transfection

To overexpress PVT1, we cloned the amplified PVT1 cDNA full‐length sequence into the pcDNA3.1 vector (Invitrogen, Carlsbad, CA, USA). To knockdown PVT1 and TRIM29, we obtained three specific short hairpin RNAs (shRNAs) for PVT1 and TRIM29 from GenePharma (Shanghai, China). The miR‐3619‐5p mimic, inhibitor, and corresponding negative control were purchased from RiboBio (Guangzhou, China). HCT116 cells were randomly divided into control, sh‐NC, sh‐PVT1, miR‐3619‐5p mimic, and Co‐OV (overexpressing PVT1 and miR‐3619‐5p together) groups. LoVo cells were randomly divided into control, pcDNA3.1‐NC, PVT1, sh‐TRIM29, and Co‐KD (knockdown miR‐3619‐5p and TRIM29 together) groups. Subsequently, according to the Lipofectamine 2000 kit instructions (Thermo Fisher Scientific, Waltham, MA, USA), all plasmids and oligonucleotides were transfected into HCT116 or LoVo cells following the grouping information. After 24 h of transfection, transfection efficiency was determined by RT‐qPCR or Western blotting.

### 2.3 RT‐qPCR assay

TRIzol reagent (TaKaRa, Tokyo, Japan) was used to extract total RNA from cells or tissues following the manufacturer's instructions. NanoDrop ND‐1000 (Thermo Fisher Scientific) was used to measure the RNA concentration. Using the PrimeScript RT kit (TaKaRa), 500 ng of total RNA was reverse transcribed into cDNA. Referring to the SYBR Premix Ex Taq kit instructions (TaKaRa), glyceraldehyde‐3‐phosphate dehydrogenase (GAPDH) or U6 was used as an internal control, and RT‐qPCR was performed using an ABI 7500 real‐time fluorescent quantitative PCR system (Applied Biosystems, USA) to detect the relative expression of PVT1 and miR‐3619‐5p. The 2^−ΔΔCt^ method was used to calculate the relative expression levels. The primer sequences are shown in Table 1. All experiments were repeated thrice.

**TABLE 1:** Primer sequence for RT‐qPCR.

Name	Forward primer (5′‐3′)	Reverse primer (5′‐3′)
PVT1	GGGTGACCTTGGCACATACA	GTCCGTCCAGAGTGCTGAAA
GAPDH	GCAACTAGGATGGTGTGGCT	TCCCATTCCCCAGCTCTCATA
miR‐3619‐5p	CATCTTTGCACTCAGCAGGC	CGTCCTTACCCCACAGCAG
U6	CCCTTCGGGGACATCCGATA	TTTGTGCGTGTCATCCTTGC

Abbreviations: GAPDH, glyceraldehyde‐3‐phosphate dehydrogenase; PVT1, plasmacytoma variant translocation 1; RT‐qPCR, reverse transcription‐quantitative polymerase chain reaction.

### 2.4 Western blotting assay

After collecting HCT116 or LoVo cells, radioimmunoprecipitation assay buffer (RIPA) lysate (Beyotime, Shanghai, China) was used to extract the total protein. A BCA kit (Beyotime) was used to measure the total protein concentration in HCT116 and LoVo cells. Proteins were separated by 10% polyacrylamide gel electrophoresis and transferred to a polyvinylidene fluoride (PVDF) membrane. About 5% bovine serum albumin (BSA) was added to the PVDF membrane for blocking, and the membrane was incubated at room temperature for 1 h. The PVDF membrane was then incubated with diluted rabbit monoclonal antibody at 4°C overnight. The next day, goat anti‐rabbit IgG H&L (HRP) was added and incubated at room temperature. Enhanced chemiluminescence (ECL) reagent (Beyotime) was used for development, and images were observed using a ChemiDoc XRS^+^ gel imaging system (Bio‐Rad, Hercules, CA, USA). GAPDH was used as an internal reference, and the average relative protein expression was expressed as the gray value of the target protein relative to GAPDH. ImageJ software (National Institutes of Health, USA) was used to analyze the gray value of the target protein bands. All antibodies were purchased from Abcam (Cambridge, MA, USA). The antibody information is shown in Table 2.

**TABLE 2:** Antibody information for Western blotting and IF.

Antibody	Cat. No.	Dilution ratio for WB	Dilution ratio for IF
Primary antibody			
E‐cadherin	ab40772	1:10000	1:500
N‐cadherin	ab18203	1:1000	1:500
Vimentin	ab92547	1:1000	1:250
Slug	ab63568	1:500	1:500
Second antibody			
Goat anti‐rabbit IgG H&L	ab7090	1:10000	1:1000

Abbreviations: IF, immunofluorescence; WB, Western blotting.

### 2.5 Cell proliferation assay

The proliferation of HCT116 and LoVo cells was analyzed using the cell counting kit‐8 (CCK‐8) kit. Briefly, 1000 cells were seeded in a 96‐well plate with complete medium and cultured for 24, 48, and 72 h. We added 10 μL of CCK‐8 for each well and incubate incubated at 37°C for 4 h. The cell absorbance at each time point was measured using a Multiskan FC microplate reader (Thermo Fisher Scientific) at a wavelength of 450 nm.

### 2.6 RNA immunoprecipitation assay

The Magna RIPTM RNA‐binding protein immunoprecipitation kit (Millipore, USA) was used to perform RNA immunoprecipitation (RIP) to analyze whether PVT1 pulled down the endogenous miR‐3619‐5p. Briefly, HCT116 cell lysates were incubated in RIP buffer containing magnetic beads coupled with human anti‐AGO2 antibody (Merck Millipore, Billerica, MA, USA). The protein was digested with proteinase K to obtain immunoprecipitated RNA. Subsequently, RT‐qPCR was performed to detect the precipitated RNA.

### 2.7 RNA pull‐down assay

Biotin RNA Labeling Mix and T7 RNA polymerase (Roche, Basel, Switzerland) were used to label miR‐3619‐5p in vitro and transfected into HCT116 cells. After 24 h, the HCT116 cells were lysed, and the collected lysate was combined with M‐280 streptavidin beads (Sigma‐Aldrich) pre‐coated with RNAse‐free BSA and yeast tRNA (Sigma‐Aldrich, St. Louis, MO, USA) and incubated at 4°C for 3 h. Then, the cells were washed twice with pre‐cooled lysis buffer, thrice with low‐salt buffer, and once with high‐salt buffer. As mentioned earlier, after the TRIzol kit extracted the total RNA, RT‐qPCR was used to detect PVT1 enrichment.

### 2.8 Invasion, migration, and colony formation assay

Transwell and wound healing assays were used to detect the invasion and migration of HCT116 and LoVo cells, respectively. For Transwell, the Transwell (Corning Incorporated, NY, USA) was placed in a 24‐well plate, and the upper chamber of the Transwell was coated with diluted Matrigel (1:8, BD Biosciences, San Jose, CA, USA). The cell density was adjusted to 1 × 10^5^ cells/mL, and the cells were inoculated into the upper chamber. DMEM (600 μL) containing 10% FBS was added to the lower chamber. After 24 h of culture, cells in the lower chamber were fixed with 4% paraformaldehyde for 15 mins and stained with 0.5% crystal violet solution for 15 mins.

An inverted microscope was used to observe and capture images, and ImageJ software was used for counting. For the wound healing assay, the cells were cultured in a six‐well plate until they reached 80%–90% confluence. Then, a 100 μL sterile pipette tip was used to scratch the cells in a straight line, and the medium was replaced with a serum‐free medium. After 24 h of initial scratching, a microscope was used to record the images and calculate the wound healing percentage (compared to 0 h). For the colony formation assay, cells were adjusted at 1000/well seeded in 6‐well plates and cultured for 2 weeks. Then, 4% paraformaldehyde was used for fixation, and crystal violet was used to stain the cells. A light microscope and ImageJ software were used to analyze the number of clones.

### 2.9 Immunofluorescence staining

HCT116 or LoVo cells that reached 80% confluence in a 24‐well culture plate were fixed with 4% paraformaldehyde for 20 mins. After penetrating the cell membrane with 0.1% Triton X‐100 for 10 mins, the antigen was blocked with 10% goat serum for 30 mins at room temperature. The cells were incubated with rabbit monoclonal antibodies against E‐cadherin, *N*‐cadherin, vimentin, and slug at 4°C overnight. The cells were then incubated with goat anti‐rabbit IgG H&L (HRP) at room temperature for 1 h, and the DNA was labeled with 4′,6‐diamidino‐2‐phenylindole for 10 mins. A fluorescence microscope was used to observe and capture images, and the fluorescence intensity was analyzed through ImageJ software. All antibodies were purchased from Abcam, and the antibody information is displayed in Table 2.

### 2.10 In vivo mice assays

Thirty‐six 6‐week‐old female BALB/c athymic nude mice were purchased from the Kunming Institute of Zoology, Chinese Academy of Sciences. As mentioned previously,^25^ HCT116 cells successfully transfected with sh‐PVT1 and negative control (about 1 × 10^6^ cells/mouse) were injected subcutaneously, tail vein injection or intrasplenic injection to induce tumor growth, lung metastasis or liver metastasis in vivo. The endpoint of the in vivo tumor growth experiment was based on weight loss, tumor volume greater than 4 cm^3^, and reduced vigorous movements. The endpoint of the in vivo liver metastasis experiment was based on clinical signs of liver metastasis, weight loss, ascites, and energy reduction behavior. The tumor growth model's tumor volume was measured at 5, 10, 15, 20, and 25 days. The mice were euthanized at 30 days after injection, and tumors, lungs and liver were collected for hematoxylin–eosin and immunohistochemistry staining analysis.

### 2.11 Statistical analysis

SPSS 21.0 software (IBM Corp., Armonk, NY, USA) was used for data analysis. The data are expressed as mean ± standard deviation, and each experiment was performed at least thrice. A one‐way analysis of variance (ANOVA) was used for multiple group comparisons. The comparison between the two groups were performed using a *t*‐test. The χ^2^‐square test determined the importance of clinicopathological parameters in patients with CRC. The Pearson's correlation coefficient was used for correlation analysis. *p* < .05 was considered statistically significant.

## 3 RESULTS

### 3.1 PVT1 was highly expressed in CRC tissues and cell lines

**FIGURE 2:**
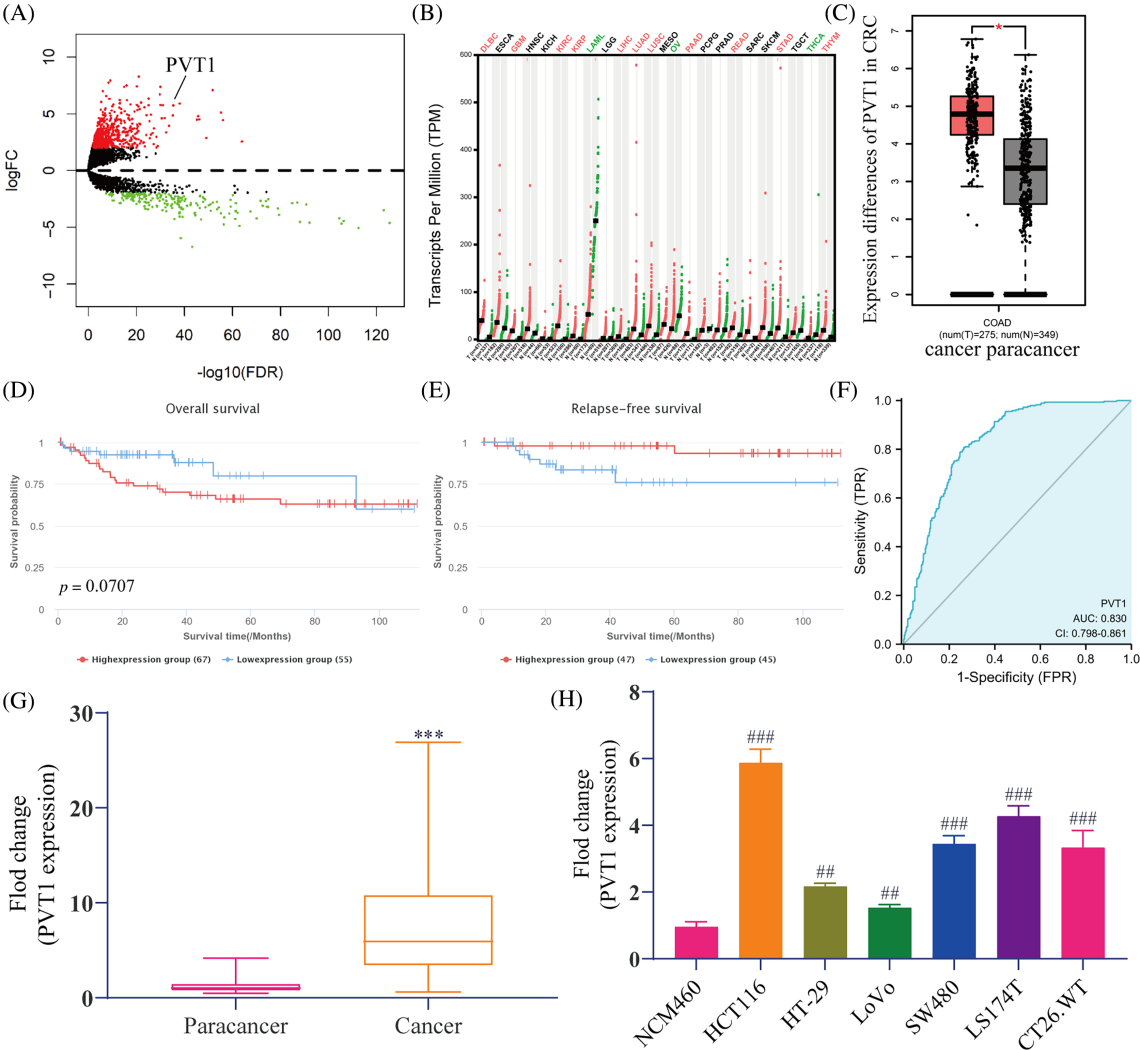
PVT1 was highly expressed in CRC tissues and cell lines. (A) Volcano plot of lncRNA difference between CRC and normal adjacent tissue. The GEPIA database predicted the expression differences of PVT1 in (B) multiple malignant tumors and (C) CRC. Red indicates high PVT1 expression in cancer; green indicates low expression; black indicates no significant difference. The lnCAR database predicted the correlation between PVT1 expression and (D) overall and (E) relapse‐free survival in patients with CRC. (F) PVT1‐related ROC curve model based on TCGA. RT‐qPCR was used to detect differences in the expression of PVT1 in (G) tissues (CRC tissues and paired adjacent tissues) and (H) cell lines (CRC cell lines and normal colonic epithelial cells). The data are presented as the mean ± SD. Student's *t*‐test; ***p < .001 versus Para cancer (*n* = 27); ^##^ *p* < .01, ^###^ *p* < .001 versus NCM460 (*n* = 3). CRC, colorectal cancer; GEPIA, gene expression profiling interactive analysis; lnCAR, lncRNAs from cancer arrays; lncRNA, long noncoding RNA; PVT1, plasmacytoma variant translocation 1; ROC, receiver operating characteristic; RT‐qPCR, reverse transcription‐quantitative polymerase chain reaction; TCGA, the Cancer Genome Atlas.

Based on the TCGA dataset, we analyzed the differentially expressed lncRNAs in CRC. As presented in Figure 2A, we found 693 up‐regulated and 200 down‐regulated lncRNAs (adjusted *p‐*value <.05 and absolute logFoldChange >2). Through gene expression profiling interactive analysis (GEPIA)^26^, we found that PVT1 was abnormally highly expressed in most malignant tumors (Figure 2B) and was the same in CRC (Figure 2C). We then analyzed the correlation between PVT1 expression and survival of patients with CRC. A comprehensive resource for lncRNAs from cancer arrays (lnCAR)^27^ analysis showed that high expression of PVT1 predicted that the overall survival of patients with CRC was reduced (Figure 2D), and the relapse‐free survival was higher (Figure 2E). Moreover, we prepared the receiver operating characteristic (ROC) curve and found that the area under the curve (AUC) of PVT1 in distinguishing CRC samples from normal samples was 0.830, indicating a great prediction model (Figure 2F). Furthermore, we verified PVT1 expression in clinical samples. The results revealed that PVT1 expression in CRC tissues was significantly higher than in corresponding adjacent tissues (Figure 2G). Similarly, PVT1 expression in CRC cell lines (HTC116, HT‐29, LoVo, SW480, LS174T, and CT26.WT cells) was significantly higher than that of normal colonic epithelial cells NCM460 (Figure 2H), and the expression was the highest in HCT116 cells and the lowest in LoVo cells. As indicated in Table 3, high PVT1 expression was related to the late tumor, node, metastasis staging, and metastasis. These findings confirmed that PVT1 is abnormally highly expressed in CRC and may be linked to CRC metastasis.

**TABLE 3:** The clinic‐pathological factors of 27 CRC patients.

Characteristics	Number of cases	PVT1 expression		*p* value
		Low (*n* = 15)	High (*n* = 12)	
Gender				
Female	14	5	9	.288^ns^
Male	13	7	6	
Age (year)				
< 60	12	5	7	.552^ns^
≥ 60	15	7	8	
Tumor site				
Colon	17	8	9	.519^ns^
Rectum	10	4	6	
Tumor invasion depth				
T1‐2	9	8	1	*.002* **
T3‐4	18	4	14	
Lymph node metastasis				
N0	16	11	5	*.003* **
N1‐2	11	1	10	
Distant metastasis				
M0	17	12	5	*.000* ***
M1	10	0	10	
TNM stage				
I + II	18	12	6	*.001* ***
III + IV	6	0	9	

*Note*: *p* < .05 was considered statistically significant (in italics). ^ns^ *p* >.05.

Abbreviations: CRC, colorectal cancer; PVT1, plasmacytoma variant translocation 1; TNM, tumor, node, metastasis.

** *p* < .01.

*** *p* < .001.

### 3.2 PVT1 promoted proliferation and EMT of CRC cells

**FIGURE 3:**
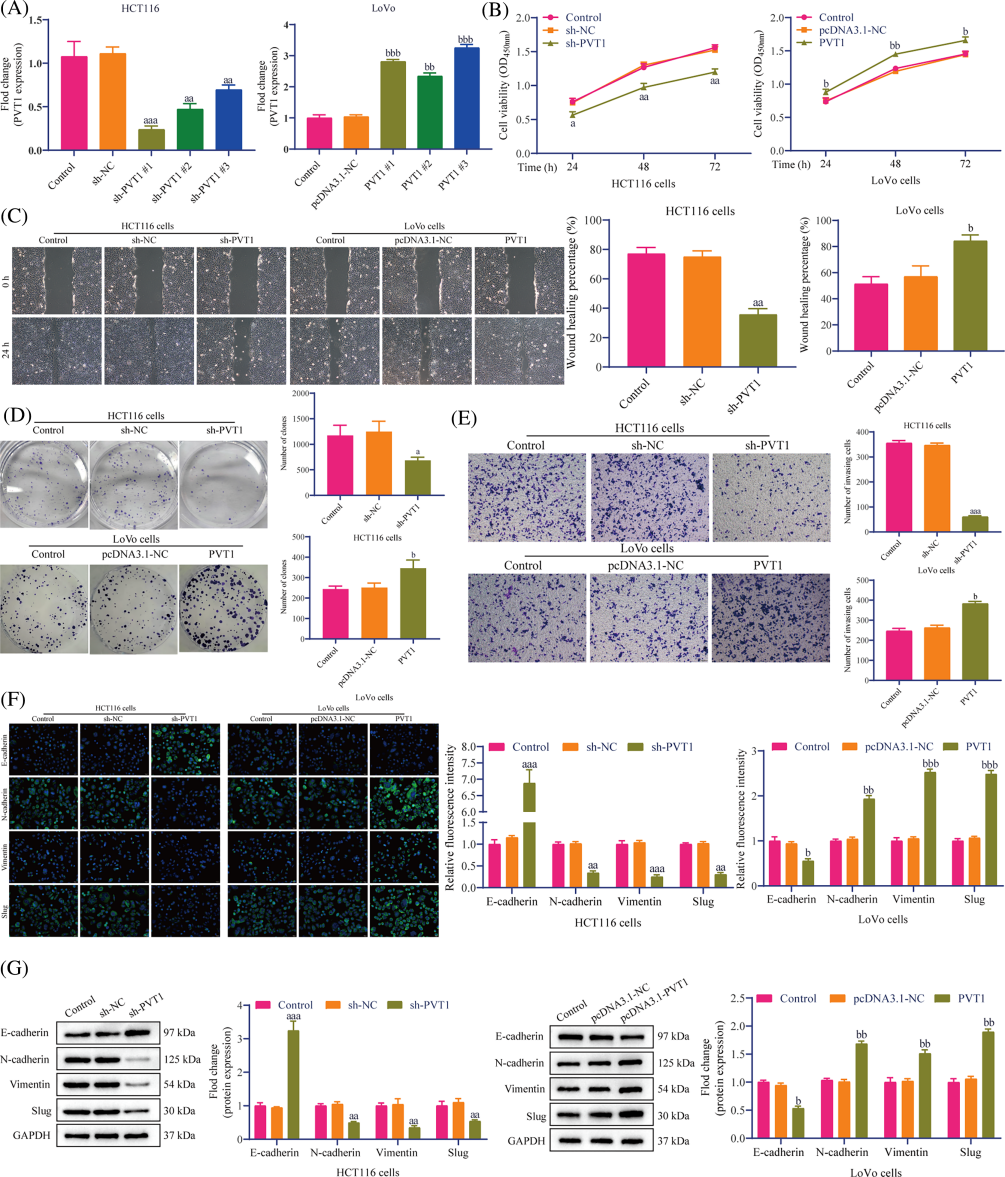
PVT1 promoted proliferation and EMT of CRC cells. (A) RT‐qPCR exhibited the transfection efficiency of sh‐PVT1 and pcDNA3.1‐PVT1. (B) The CCK‐8 kit was used to analyze the effect of PVT1 on HCT116 and LoVo cell proliferation. (C) The wound healing assay was used to detect the wound healing rate of CRC cells after 24 h. Scale bar: 100 μm. (D) Colony formation and (E) Transwell assays were performed to detect CRC cells' colony and invasion ability. Scale bar: 100 μm. (F) IF staining showed the localization and fluorescence intensity of EMT‐related proteins (E‐cadherin, *N*‐cadherin, vimentin, and slug) in HCT116 and LoVo cells. Scale bar: 20 μm. (G) Expression of EMT‐related proteins was detected by Western blotting. All in vitro data are representative of three independent experiments. The data are presented as the mean ± SD. Student's *t*‐test; ^a^ *p* <.05, ^aa^ *p* <.01, ^aaa^ *p* <.001 versus sh‐NC; ^b^ *p* <.05, ^bb^ *p* <.01, ^bbb^ *p* <.001 versus pcDNA3.1‐NC. CCK‐8, cell counting kit‐8; CRC, colorectal cancer; EMT, epithelial‐mesenchymal transition; IF, immunofluorescence; PVT1, plasmacytoma variant translocation 1; RT‐qPCR, reverse transcription‐quantitative polymerase chain reaction.

To explore whether the high PVT1 expression is related to CRC metastasis, we attempted to confirm the function of PVT1 in the proliferation and metastasis of CRC cells. We transfected sh‐PVT1 and pcDNA3.1‐PVT1 into HCT116 and LoVo cells, respectively. RT‐qPCR results showed that sh‐PVT1 transfection significantly down‐regulated PVT1 expression in HCT116 cells. After pcDNA3.1‐PVT1 transfection, the PVT1 expression in LoVo cells was up‐regulated considerably (Figure 3A). We chose the best sh‐PVT1 #1 and pcDNA3.1‐PVT1 #2 for the follow‐up experiments. The CCK‐8 results indicated that, compared to the control group, PVT1 knockdown prevented the growth of HTC116 cells. Concurrently, PVT1 overexpression promoted LoVo cells proliferation (Figure 3B). Moreover, we explored the effect of PVT1 on the metastasis of HTC116 and LoVo cells. The wound healing assay showed that in HCT116 cells, compared to the control group, the wound healing rate of the sh‐PVT1 group was significantly lower. However, in LoVo cells, the PVT1 group wound healing rate was significantly higher than the control group (Figure 3C). Similarly, colony formation (Figure 3D) and Transwell assays (Figure 3E) showed that PVT1 knockdown significantly reduced the cell colony formation, and the number of invasive cells in CRC cells, while PVT1 overexpression had the opposite effect. We further tested the expression levels of the EMT‐related proteins. IF results showed that PVT1 knockdown could enhance the fluorescence intensity of E‐cadherin in HCT116 cells and reduce the N‐cadherin, vimentin, and slug (Figure 3F). PVT1 overexpression resulted in the opposite effect in LoVo cells (Figure 3F). Western blotting showed consistent results (Figure 3G). It is suggested that PVT1 can promote CRC cells' proliferation, migration, invasion, and EMT.

### 3.3 PVT1 promoted CRC tumorigenesis and metastasis in vivo

**FIGURE 4:**
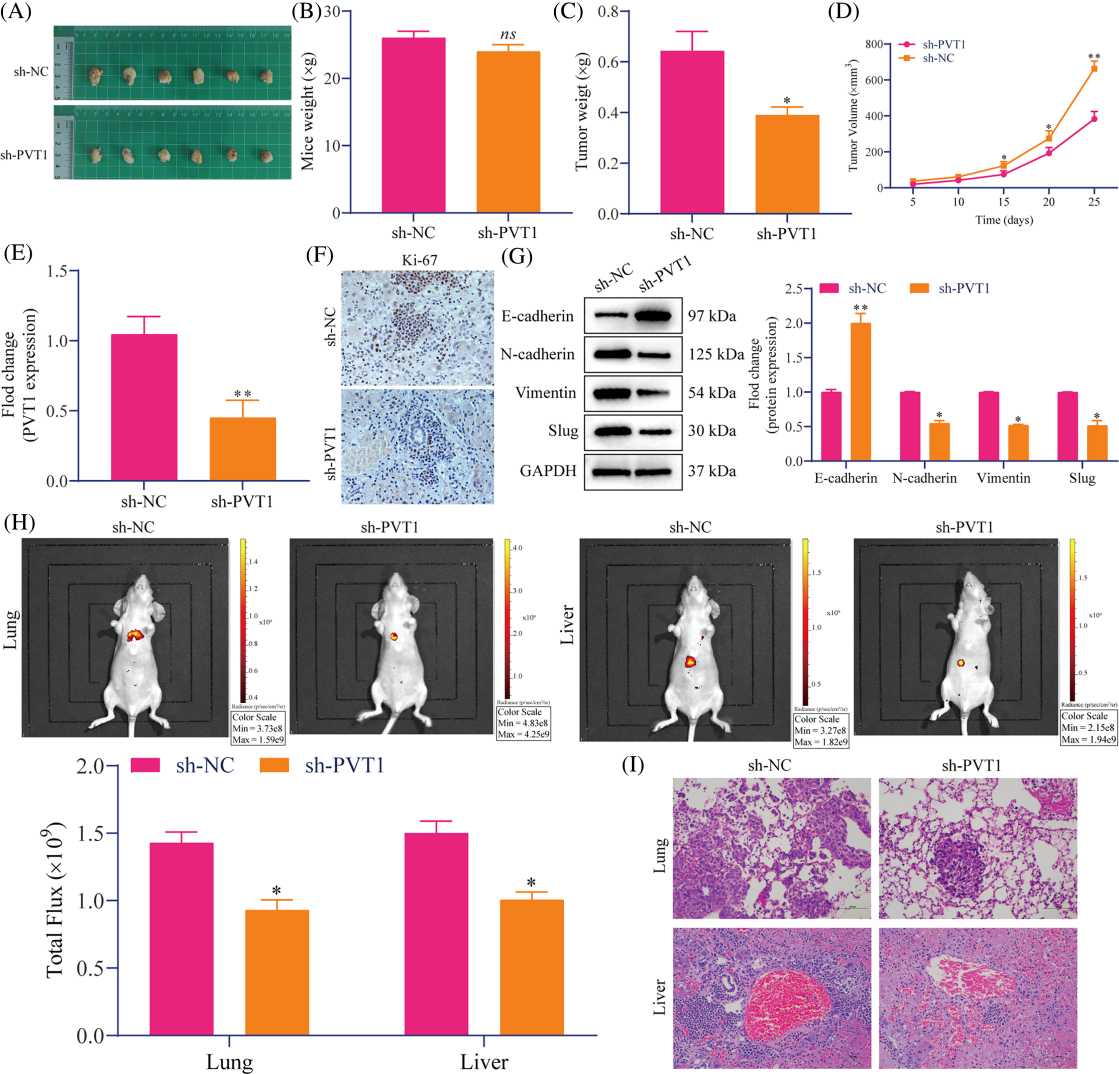
PVT1 promoted CRC tumorigenesis and metastasis in vivo. (A) A schematic diagram of the tumor obtained by subcutaneously injecting HTC116 cells transfected with sh‐NC or sh‐PVT1 into nude mice. (B) Tumor weight, (C) mice weight, and (D) tumor volume change in tumor growth model. (E) RT‐qPCR was used to detect the PVT1expression level in tumors (F) IHC staining exhibited changes in Ki‐67 expression in tumors. Scale bar: 20 μm. (G) Western blotting indicated the EMT‐related protein expression in tumors. (H) Bioluminescence images of lung and liver metastasis models were constructed using the tail vein and intrasplenic injections, respectively. (I) Metastatic nodules were detected by HE staining in the lung and liver metastasis models. Scale bar: 20 μm. Data are presented as the mean ± SD. All in vivo data are representative of six independent experiments. Student's *t*‐test; *p < .05, **p < .01, versus sh‐NC; ns indicates no significant difference. CRC, colorectal cancer; HE, hematoxylin–eosin; IHC, immunohistochemistry; PVT1, plasmacytoma variant translocation 1; RT‐qPCR, reverse transcription‐quantitative polymerase chain reaction.

We further constructed tumor growth, lung metastasis, and liver metastasis models to explore the effect of PVT1 on tumorigenicity and metastasis of CRC in vivo. Figure 4A shows the tumors of mice in the sh‐NC and sh‐PVT1 groups at 25 days. The analysis found that the volume and weight of the tumors in the sh‐PVT1 group were significantly lower than those in the sh‐NC group (Figure 4B,D), and there was no significant difference in the mice weight of the two groups (Figure 4C). This shows that PVT1knockdown inhibits CRC cell growth in vivo. RT‐qPCR results showed that the expression of PVT1 in the sh‐PVT1 group was significantly lower than that in the sh‐NC group (Figure 4E). In addition, the proportion of positive Ki‐67 cells decreased in the sh‐PVT1 group (Figure 4F). Western blotting results demonstrated that PVT1 knockdown increased E‐cadherin and reduced *N*‐cadherin, vimentin, and slug expression in tumors (Figure 4G). We constructed CRC lung and liver metastasis models to confirm whether PVT1 affects CRC metastasis in vivo, and tracked the metastasis dynamics using quantitative bioluminescence imaging. The results revealed that lung and liver metastases formed in mice injected with sh‐PVT1 HTC116 cells were significantly lower than in the sh‐NC group (Figure 4H). HE staining of the lung and liver showed that the number of metastatic nodules in the sh‐PVT1 group was markedly lower than that in the sh‐NC group (Figure 4I). It is suggested that PVT1 knockdown can inhibit the tumorigenicity and metastasis of CRC cells in vivo.

### 3.4 PVT1 acts as a ceRNA by sponging miR‐3619‐5p to regulate TRIM29 expression

**FIGURE 5:**
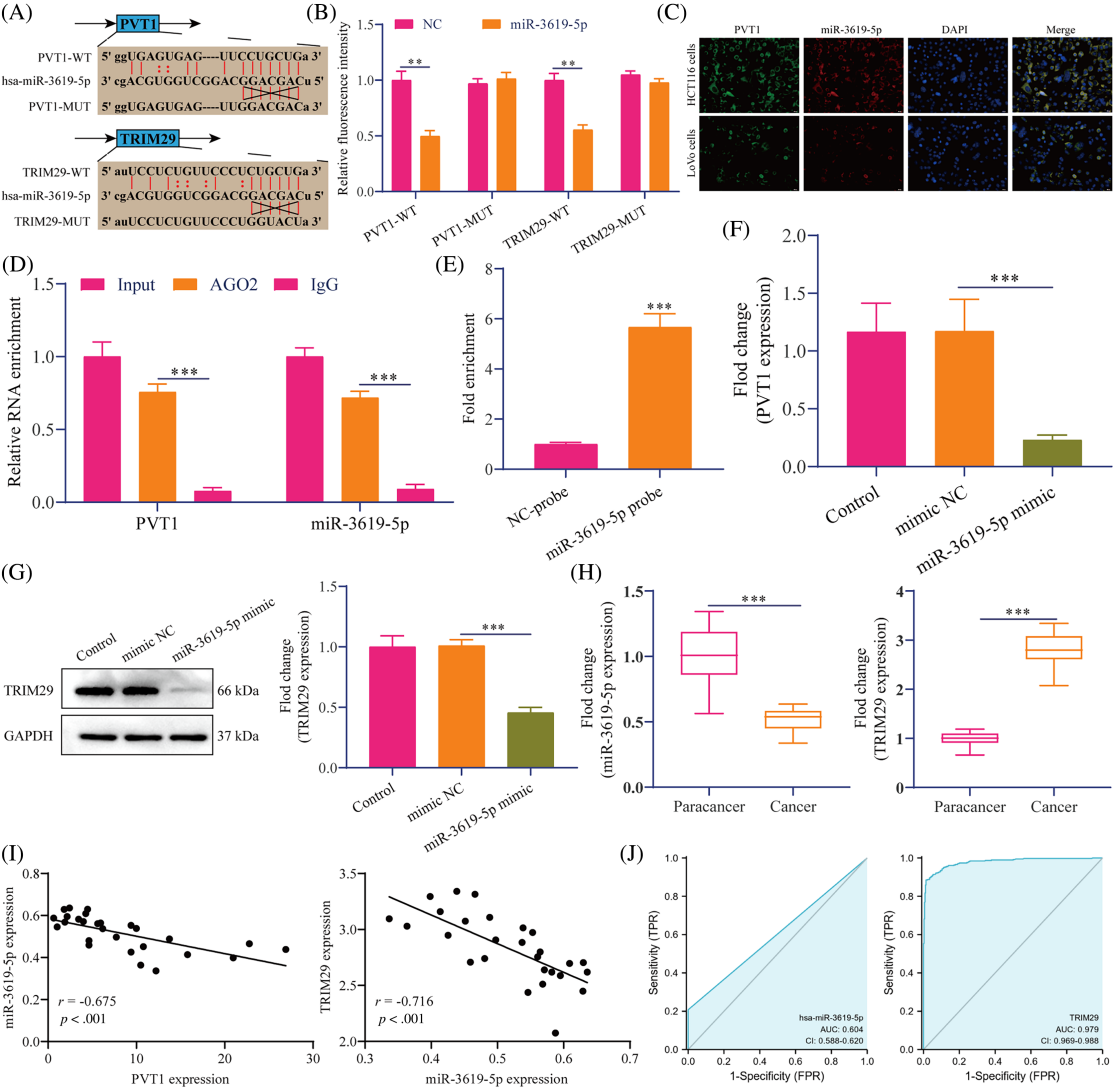
PVT1 acts as a ceRNA by sponging miR‐3619‐5p to regulate TRIM29 expression. (A) The schematic diagram showed potential binding sequences using the Starbase database. (B) The dual‐luciferase reporter gene detected the luciferase activity. (C) FISH staining showed the localization in cells. Scale bar: 20 μm. **p < .01 versus NC. (D) RIP assay. ***p < .001 versus AGO2. (E) PVT1 level of the streptavidin‐captured part in the HTC116 cell lysate. ***p < .001 versus NC‐probe. RT‐qPCR (F) and Western blotting (G) were performed to detect PVT1 and TRIM29 expression, respectively. ***p < .001 versus mimic NC. (H) Detection of TRIM29 and miR‐3619‐5p expression using RT‐qPCR. **p < .01, ***p < .001 versus Para cancer. (I) Pearson's correlation analysis. (J) The ROC curve model was constructed based on TCGA data. All in vitro data are representative of three independent experiments. The data are presented as the mean ± SD. Student's *t*‐test. ceRNA, competitive endogenous RNA; FISH, fluorescence in situ hybridization; PVT1, plasmacytoma variant translocation 1; RIP, RNA immunoprecipitation; ROC, receiver operating characteristic; RT‐qPCR, reverse transcription‐quantitative polymerase chain reaction; TCGA, the Cancer Genome Atlas; TRIM29, tripartite motif‐containing 29.

We further explored the potential downstream targets of PVT1. As presented in Figure 5A, PVT1 and miR‐3619‐5p, miR‐3619‐5p, and TRIM29 have potential binding sequences predicted by Starbase.^28^ We performed a dual‐luciferase reporter gene assay after mutating the 3′UTR region of PVT1 and TRIM29. The results showed that in 293 T cells, miR‐3619‐5p overexpression significantly inhibited the luciferase activity of PVT1 and TRIM29 wild‐type vectors and had no significant effect on the luciferase activity of mutant vectors (Figure 5B). Since PVT1 and TRIM29 bind to miR‐3619‐5p with similar 3′UTR sequences. Fluorescence in situ hybridization was used to locate PVT1 and miR‐3619‐5p in HCT116 and LoVo cells. As demonstrated in Figure 5C, PVT1 and TRIM29 were colocalized in HCT116 and LoVo cell cytoplasm. Next, we performed RIP analysis using an AGO2 antibody in HCT116 cells. Our results showed that the AGO2 antibody significantly enriched PVT1 and miR‐3619‐5p (Figure 5D). To verify the interaction between PVT1 and miR‐3619‐5p further, we designed a specific biotinylated miR‐3619‐5p probe to perform RNA pull‐down assay. The results showed that biotinylated miR‐3619‐5p effectively captured PVT1 (Figure 5E). RT‐qPCR and Western blotting results showed that miR‐3619‐5p overexpression significantly inhibited the expression of PVT1 and TRIM29 in HCT116 cells (Figure 5F,G). These results indicated that PVT1 acts as a ceRNA by sponging miR‐3619‐5p to regulate TRIM29 expression. Moreover, we detected miR‐3619‐5p and TRIM29 expression in clinical samples. The results showed that miR‐3619‐5p was significantly under‐expressed in CRC tissues, while TRIM29 was the opposite (Figure 5H). Pearson correlation analysis revealed that the expression levels of PVT1 and miR‐3619‐5p, miR‐3619‐5p, and TRIM29 in CRC tissues were negatively correlated (Figure 5I). The ROC curve showed that the AUCs of miR‐3619‐5p and TRIM29 when distinguishing CRC samples from normal samples were 0.604 and 0.979, respectively, indicating that the model had a good predictive effect (Figure 5J). These data suggest that PVT1 likely regulates CRC metastasis by regulating the miR‐3619‐5p/TRIM29 axis.

### 3.5 PVT1 promoted proliferation and EMT of CRC cells via miR‐3619‐5p/ TRIM29 axis

**FIGURE 6:**
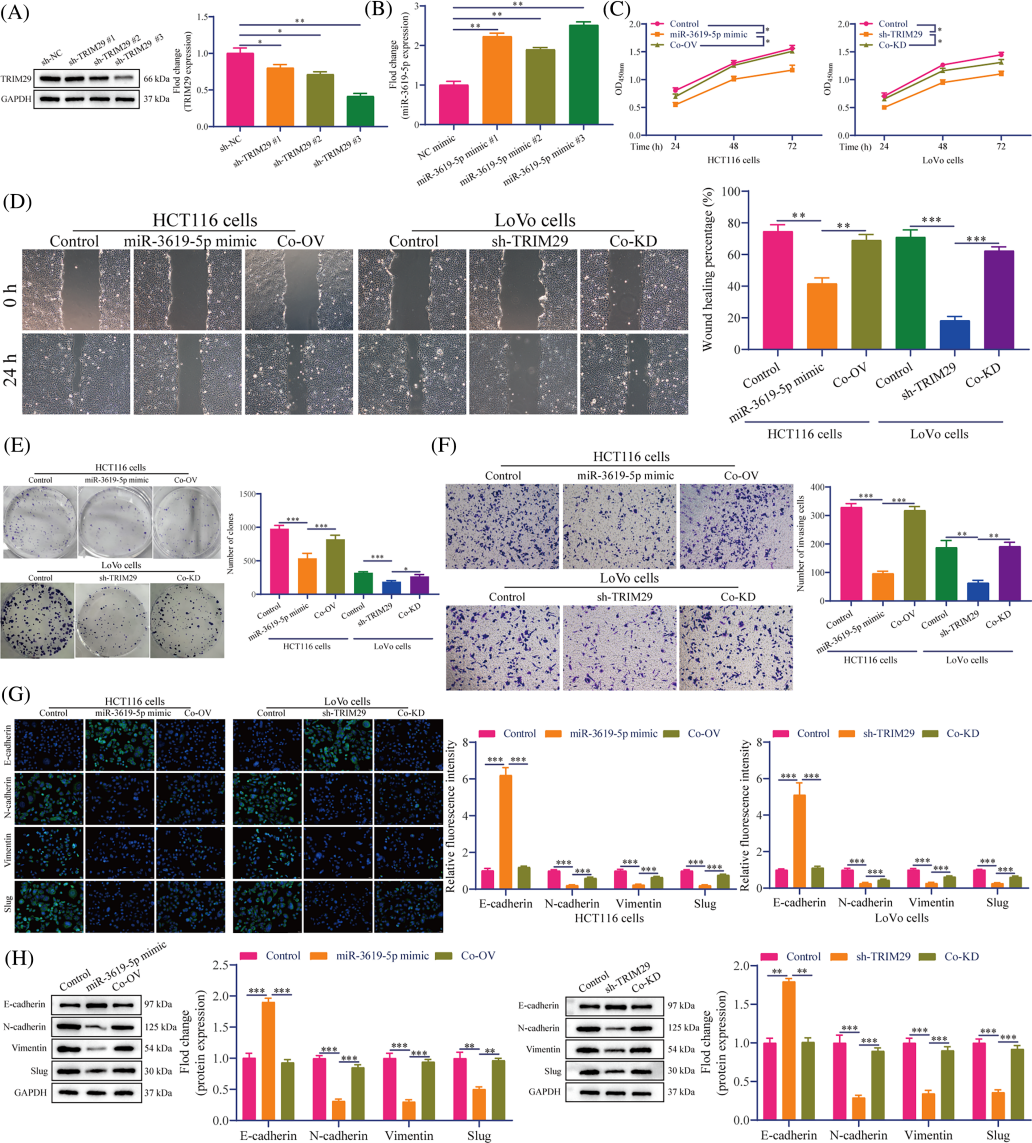
PVT1 promoted proliferation and EMT of CRC cells via the miR‐3619‐5p/ TRIM29 axis. Transfection efficiency was detected by Western blotting (A) and RT‐qPCR (B), respectively. (C) The CCK‐8 assay indicated proliferation. (D) Wound healing assay. Scale bar: 100 μm. (E) Colony formation and (F) Transwell assay showed the clones and invasive cells. Scale bar: 100 μm. (G) IF staining. Scale bar: 20 μm. (H) Western blotting was performed to detect the expression of E‐cadherin, *N*‐cadherin, vimentin, and slug proteins. All in vitro data are representative of three independent experiments. The data are presented as the mean ± SD. Student's *t*‐test; *p < .05, **p < .01, ***p < .001 versus sh‐NC. CCK‐8, cell counting kit‐8; CRC, colorectal cancer; EMT, epithelial‐mesenchymal transition; IF, immunofluorescence; PVT1, plasmacytoma variant translocation 1; RT‐qPCR, reverse transcription‐quantitative polymerase chain reaction; TRIM29, tripartite motif‐containing 29.

Finally, we verified the effect of the PVT1/miR‐3619‐5p/TRIM29 molecular axis on CRC cell metastasis in vitro. RT‐qPCR and Western blotting results showed that miR‐3619‐5p mimic transfection in HCT116 cells and sh‐TRIM29 transfection in LoVo increased miR‐3619‐5p expression and reduced TRIM29 expression (Figure 6A,B). CCK‐8 results showed that miR‐3619‐5p overexpression and TRIM29 knockdown inhibited HCT116 and LoVo cell proliferation, whereas PVT1 and miR‐3619‐5p overexpression or simultaneous miR‐3619‐5p and TRIM29 knockdown rescued CRC cell proliferation (Figure 6C). The wound healing assay results showed that, compared with the control group, the wound healing rate in the miR‐3619‐5p mimic and sh‐TRIM29 groups was significantly reduced. The Co‐OV and Co‐KD groups were significantly increased (Figure 6D). Colony formation and Transwell assays showed that miR‐3619‐5p overexpression and TRIM29 knockdown reduced the colony formation and number of invasions of CRC cells, respectively (Figure 6E,F). As expected, miR‐3619‐5p overexpression and TRIM29 knockdown significantly increased the fluorescence intensity of E‐cadherin in CRC cells and decreased *N*‐cadherin, vimentin, and slug (Figure 6G). Simultaneous of PVT1 and miR‐3619‐5p overexpression or miR‐3619‐5p and TRIM29 knockdown rescued the levels of E‐cadherin, *N*‐cadherin, vimentin, and slug (Figure 6G). Western blotting showed consistent results (Figure 6H). It is suggested that the effect of PVT1 on the proliferation and metastasis of CRC cells is achieved by sponging miR‐3619‐5p to up‐regulate the TRIM29 expression.

## 4 DISCUSSION

The incidence and mortality of CRC are increasing annually, and the incidence increased by 25% between 2007 and 2017.^29^ According to statistics, there have been 1.8 million cases of CRC and 876 000–916 000 deaths in 2017. In 2017, CRC caused 19 million disability‐adjusted life‐years, 95% of which came from years of life lost and 5% from years lived with disabilities.^29^ The CRC's poor prognosis is largely related to the distant metastasis of cancer cells, which accounts for about 90% of cancer‐related deaths.^30^, ^31^ This study, found that lncRNA PVT1 expression is related to the CRC prognosis, and has good diagnostic value. In vitro experiments showed that PVT1 acts as a ceRNA through sponging miR‐3619‐5p to regulate TRIM29 expression, and PVT1 promotes the proliferation, migration, invasion, and EMT of CRC cells by regulating the miR‐3619‐5p/TRIM29 axis. In vivo experiments also showed that PVT1 promotes CRC cell tumorigenesis and metastasis.

The Encyclopedia of DNA Elements (ENCODE) estimates that the human genome encodes more than 28 000 different lncRNAs.^32^ In recent years, many studies have focused on the role of multiple lncRNAs in regulating the biological behavior of malignant tumors, including proliferation, migration, expansion, immortality, angiogenesis, and tumor immunity.^33^, ^34^ Due to the complex interaction network involved in lncRNAs, the relationship between lncRNAs and tumor treatment resistance has received increasing attention. Numerous reports have described the intricate interactions between different RNA types, including mRNA and noncoding RNA, like lncRNAs, pseudogenes, and circular RNA. These RNA transcripts act as ceRNAs or natural miRNA sponges; they communicate with each other and regulate together by competing for binding to shared miRNAs. This new type of RNA crosstalk will better understand gene regulatory networks and impact human development and disease.^35^, ^36^ LncRNA PVT1 has been reported to be a key tumor gene, that promotes the growth of liver cancer by promoting cell proliferation, migration, and invasion through regulating the expression of MMP9.^37^ Moreover, lncRNA PVT1 mediated gastric cancer progression through regulating VEGFA to effect angiogenesis.^38^ Wu et al. confirmed that lncRNA PVT1 exerted a tumor‐promoting effect in CRC.^39^ Consistent with this, our results showed that PVT1 knockdown could inhibit CRC cells' proliferation, migration, invasion, and EMT. Studies have shown that many lncRNAs regulate the metastasis of various malignant tumors through the ceRNA mechanism. Wang et al. showed that STAT3‐mediated lncRNA HOXD‐AS1 acts as a ceRNA to promote hepatocellular carcinoma cell migration, invasion, and EMT in vitro by regulating SOX4, and distant lung metastasis in vivo.^40^ The lncRNAs FAM225A promotes nasopharyngeal carcinoma cell proliferation, migration, invasion, EMT, tumor growth, and metastasis by acting as a ceRNA of sponge miR‐590‐3p/miR‐1275 and up‐regulates ITGB3.^41^ LncRNA pro‐transition associated RNA regulates the ZEB1 expression by competitively binding miR‐101‐3p to promote EMT, invasion, and metastasis of serous ovarian cancer.^42^ In this study, we discovered a new ceRNA network in which PVT1 acts as a ceRNA by sponging miR‐3619‐5p to regulate TRIM29 expression, which is involved in CRC proliferation, migration, invasion, and EMT in vitro, as well as tumor growth and lung and liver metastases in vivo. EMT is a developmental process that promotes the movement of originally adhered epithelial cells. EMT and its reverse process, mesenchymal‐epithelial transformation, occur in wound healing and fibrosis in the whole organism.^43^, ^44^ However, they also endow cancer cells with malignant properties, including aggressive behavior, cancer stem cell activity, greater resistance to cancer cells, chemotherapy, and immunotherapy.^43^, ^45^ This indicates a central role of EMT in tumor cell metastasis. We confirmed that PVT1 promotes tumorigenicity, lung, and liver metastasis of CRC cells in vivo and promotes their proliferation, migration, invasion, and EMT in vitro. PVT1 has been proven to drive metastasis in various tumors, including gastric cancer,^46^ osteosarcoma,^47^ breast cancer,^48^ and gallbladder cancer.^49^ Gharib et al. showed that PVT1 can be used as a predictive indicator for screening patients with CRC with lymph node metastasis.^50^ PVT1 is a new carcinogenic promoter of MYC in CRC, and its activity is controlled by epigenetic regulation mediated by abnormal methylation.^51^ In our study, the molecular mechanism underlying PVT1 carcinogenesis was further elucidated.

This study uncovered a new ceRNA regulatory network: lncRNA PVT1/miR‐3619‐5p/TRIM29 in CRC. Moreover, functional and mechanistic analyses have determined the favorable role of PVT1 in CRC growth and metastasis. It promotes TRIM29 expression by sponging miR‐3619‐5p, leading to CRC cells' growth and metastasis in vivo and in vitro. This suggests that PVT1 is vital in the occurrence and development of CRC tumors and highlights that it can be used as a prognostic indicator and promising therapeutic target for CRC.

## AUTHOR CONTRIBUTIONS

**Zhenni Sun:** Conceptualization; validation; writing – review and editing. **Xutong Li:** Conceptualization; data curation; formal analysis; methodology; validation. **Yanyan Shi:** Methodology; software; validation; writing – original draft; supervision. **Yasai Yao:** Conceptualization; methodology; writing – review and editing; supervision.

## CONFLICT OF INTEREST STATEMENT

All authors declare that they have no conflict of interest.

## ETHICS STATEMENT

This study has been approved by the Ethics Committee of Qingdao Municipal Hospital, and all patients have signed informed consent. Batch number: XS202404007. In vivo mice assays were approved by the Ethics Committee of Qingdao Municipal Hospital.

## PATIENT CONSENT FOR PUBLICATION

All patients involved in this study indicated that they knew and agreed that this paper would be published in Cancer Reports.

## Data Availability

The data that support the findings of this study are available from the corresponding author upon reasonable request.
